# Boys with constitutional delay of growth and puberty developed spontaneous puberty and reached standard adult height without pharmacological therapy

**DOI:** 10.1016/j.jped.2025.101429

**Published:** 2025-09-19

**Authors:** Thais Milioni Luciano, Mônica Freire Stechinni, Sonir Roberto Rauber Antonini

**Affiliations:** Universidade de São Paulo (USP) Faculdade de Medicina de Ribeirão Preto (FMRP), Departamento de Puericultura e Pediatria, Divisão de Endocrinologia Infantil, Ribeirão Preto, SP, Brazil

**Keywords:** Delayed puberty, Constitutional delay of growth and puberty, Hypogonadism

## Abstract

**Objectives:**

To evaluate the patterns of pubertal development, growth, and adult height in untreated male patients with CDGP.

**Methods:**

A retrospective study was conducted at a tertiary care center from 1984 to 2019. Medical records of 46 boys diagnosed with CDGP (after excluding those with hypogonadism) were included for further analysis.

**Results:**

Most patients were born at term (78%) and appropriate for gestational age (85%). A family history of delayed puberty was noted in 50%. The median age at initial evaluation was 14.3 years (range: 4.8–16.2 years). Short stature before puberty was the main reason for seeking medical attention (48%). Short stature was common at the first evaluation (93%) but improved over time; at the final assessment, only 17% of the patients remained short. For those who reached adult height, the height Z-scores were comparable to target height. Predictions using the Bayley-Pinneau method often overestimated adult height. Delayed bone age was present in 82% of patients at initial evaluation. The median age of spontaneous pubertal onset was 15 years, with a median duration of 2.1 years (range: 1.2–4.8 years, n = 33). None of the patients received pharmacological treatment. The median age at Tanner stage G5 was 17.1 years.

**Conclusions:**

In boys with CDGP, transient short stature improved spontaneously during puberty. Most achieved their target height without growth-promoting therapy. However, adult height predictions based on the Bayley-Pinneau method were often overestimated. Spontaneous puberty initiation and completion occurred at approximately 15 and 17 years of age, respectively.

## Introduction

Puberty is characterized by a growth spurt, acquisition of sexual maturity, and reproductive function [1]. In boys, the initial signs include testicular volume ≥ 4 mL (ml) and/or Tanner stage G2. Absence of these characteristics at the age of 13.5 - 14 years old is called delayed puberty (DP) [2]. Constitutional delay of growth and puberty (CDGP) is the most common cause of DP, diagnosed after excluding pathological etiologies [2, 3, 4, 5].

CDGP patients are typically healthy, with no history of prenatal or perinatal morbidities, chronic illnesses, and eating disorders [5]. The patient's characteristics include prepubertal short stature, growth below the target height (TH), and reduced growth velocity [4, 5, 6, 7, 8]. Low body mass index (BMI), without malnutrition, and delayed adrenarche are also common [4, 5, 6, 7, 8]. The spontaneous progression of puberty is often the only way to confirm the diagnosis [8,9]. Family history of DP occurs in 50–80 % of the cases, often showing an autosomal dominant inheritance pattern [3,4,5,10]. Recent studies have explored the genetic basis of CDGP, including potential associations with polymorphisms in the androgen receptor (AR) gene, although these findings require further validation [11].

These adolescents usually present with delayed bone age, low gonadotropins, testosterone, and growth factors, such as insulin-like growth factor 1 (IGF-1), levels [8,11]. While suggestive, these findings alone do not confirm CDGP [5]. The traditional management involves expectant observation, with interventions reserved for cases of significant psychosocial impairment. For the therapy, low-dose testosterone in boys may be administered for four to six months, and aromatase inhibitors such as letrozole, can also be proposed as an alternative in selected cases of CDGP [4,5,7,9,12,13].

Although CDGP is often considered within the spectrum of normal growth variation, conflicting evidence exists regarding adult height (AH) outcomes. Some studies suggest AH aligns with TH [2]. Nevertheless, due to the frequent presentation with short stature during adolescence, some authors have proposed that CDGP may temporarily fit the diagnostic criteria for idiopathic short stature (ISS), despite its benign and self-limited nature [5]. Variability in AH outcomes underscores the need for a deeper understanding of CDGP's natural history. This study elucidated the clinical features, growth patterns, pubertal development, and AH outcomes of male CDGP patients observed over a 35-year period at a referral center.

## Methods

This retrospective cohort study was conducted at a single tertiary care center, the Ribeirão Preto Medical School, University of São Paulo (HC-FMRP-USP), located in Ribeirão Preto, São Paulo, Brazil.

Data were collected from medical records of patients followed at the general pediatric outpatient clinic for growth and development problems, and at the pediatric endocrinology outpatient clinics, between January 1984 and December 2019. Records were identified using keywords including “delayed puberty,” “pubertal delay,” “constitutional delay of growth and puberty,” “constitutional short stature,” “constitutional and familial short stature,” “late maturation,” “constitutional pubertal delay,” “hypogonadism,” “infantilism,” or “poor sexual development.”

Male patients diagnosed with DP, followed for a minimum of one year, were included. Patients with systemic chronic diseases, definitive hypogonadism, use of medications, or conditions associated with DP were excluded.

Perinatal data collected included gestational age (classified as term or preterm), birth weight, perinatal complications, and genitalia abnormalities at birth. Newborns were categorized as small for gestational age (SGA), appropriate for gestational age (AGA), or large for gestational age (LGA) based on weight Z-scores [14].

The parents' stature was collected and used to calculate TH with the formula: TH = [(maternal height + paternal height + 13) / 2] ± 10 cm [15]. History of parental DP was defined by a reported history of delayed pubertal timing in either parent and/or mother's age at menarche ≥14 years.

Follow-up data included reason for medical consultation, age at referral, height, and body mass index (BMI) at each visit, age at puberty onset (testicular volume ≥ 4 ml and/or Tanner stage G2), and age at puberty end (Tanner G5) [3,5]. In cases of lost follow-up, patients were contacted by telephone to measure adult height and weight at a primary care unit.

Bone age, determined according to the Greulich-Pyle method [16], was classified as delayed (if less than −2 SD for age and sex according to chronological age), concordant (between −2 SD and +2 SD), or advanced (greater than +2 SD). The interpretation of bone age was performed by two experienced physicians during patient follow-up. In cases where there was disagreement between the two evaluators, a third experienced physician reviewed the radiograph, and the final bone age was determined by consensus among the three evaluators.

Predicted adult height (PAH) was calculated according to bone age – which was compatible (PAHc) or delayed (PAHr) in comparison with chronological age – using the Bayley-Pinneau method [17]. The last bone age available in the records was used for this purpose, as long as it was not greater than 16 years. The same radiography was used to calculate PAHc and PAHr [17]. For the subjects whose AH was known, the differences between the Z-score of AH and TH, and of AH and PAH were calculated. Anthropometric data (height and BMI) were converted into Z-score by the WHO AnthroPlus 1.0.4 software (available for download at https: // www.who.int/growthref/tools/en/), based on WHO standardized growth charts for children and adolescents aged 5–19 years (2007) [18].

To assess height and BMI progression according to age, the correlation between age and Z-score of these measures was analyzed using Bland & Altman correlation coefficients with repeated observations. This coefficient is useful to understand the correlation between multiple observations of the same subject at different times, as well as between different subjects [19,20]. This study was approved by the Ethics Committee of HC-FMRP-USP (project Number 3.805.964), which granted a waiver of informed consent due to the retrospective nature of the study.

## Results

**Figure. 1:**
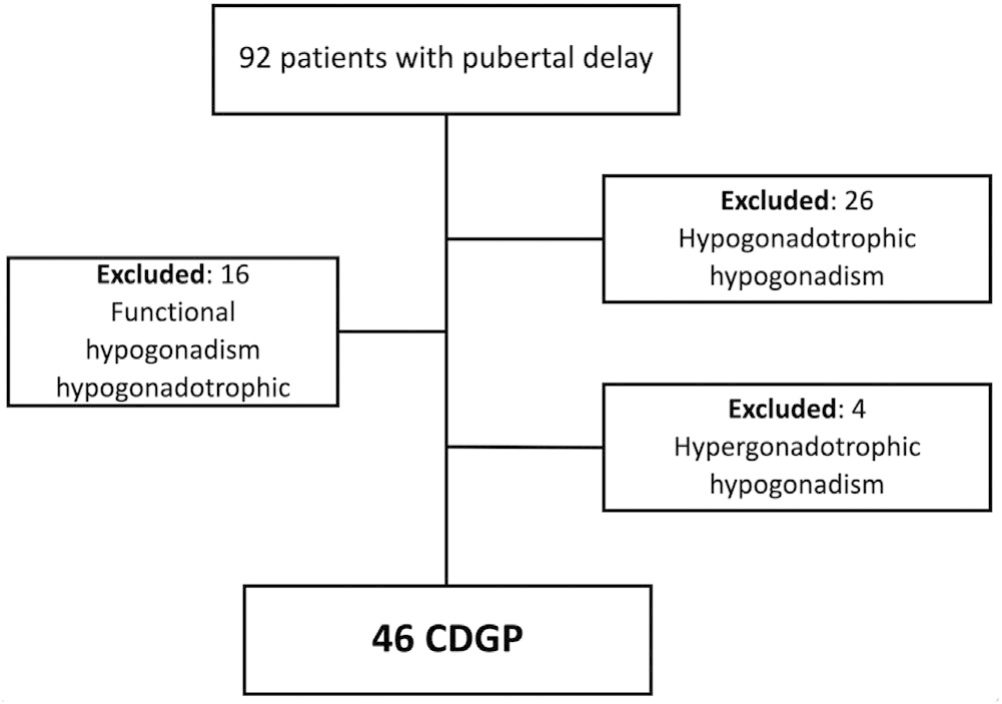
Composition of the sample. Obs: The high number of cases with hypogonadotropic hypogonadism reflects the tertiary referral profile of our center, which manages complex endocrine disorders such as congenital or acquired panhypopituitarism.

The medical records of 92 male patients with DP were reviewed. Forty-six patients were diagnosed with CDGP and were eligible for the study. Figure 1 shows the composition of the study sample. It should be noted that the high number of cases with hypogonadotropic hypogonadism likely reflects the tertiary referral profile of the center, which manages complex endocrine disorders such as congenital or acquired panhypopituitarism

At the first appointment, the median age was 14.3 years (range: 4.8 to 16.2), with 65 % of patients aged over 14 years. The most common reasons for seeking medical care were poor gain of weight and stature (48 %), absence of growth spurt (24 %), and lack of sexual characteristics development (22 %). In 6 % of the cases, DP was noted by the pediatrician but not by the patient or the family. At the last visit, the median age was 17.7 years (range: 15 to 20.7), with a median follow-up duration of 2.9 years (range: 1.1 to 13).

Most patients were born at term (78 %), and 17 % were preterm. Most patients were AGA (85 %), 2 % were LGA, and none were SGA. However, gestational age and birth weight information were not available in 5 % and 13 % of the medical records, respectively. At birth, no abnormalities in the genital exam were noted, except for unilateral cryptorchidism in one patient. In two patients, information on the external genitalia was missing. Except for prematurity in some cases, no patient had other neonatal complications or malformations.

A family history of CDGP was positive in 50 % of cases, negative in 41 %, and unavailable in 9 % of the medical records. Information about maternal age at menarche was available in only 14 cases; in nearly 70 % of the records, this information was not documented. Among the records where maternal menarche was reported, menarche was typically timed in 4 cases and delayed in 10 cases. Data regarding paternal puberty were available for only 5 of the 46 patients. In two cases, the reported pubertal milestone was the growth spurt, which occurred at ages 13 and 14 years. In the remaining three cases, the milestone reported was the onset of shaving, which occurred at age 16 in one case and at age 22 in two cases.

The first visit to the specialist occurred at varying ages between 11 and 15 years. At that time, 93 % of the patients indeed presented with short stature, with a median height Z-score of −2.4 SD, ranging from −3.4 to 0.8 SD. At the last visit, which occurred on average between ages 16 and 18, even when growth velocity indicated that final height had not yet been reached, only 17 % of the patients remained with short stature. The median height Z-score had increased to −1.3 SD, ranging from −2.8 to 0.8 SD. Maternal and paternal Height and height Z-Score, as well as TH and AH, are summarized in Table 1.

**Table 1:** Parental height, target height, and patient’s adult height.

Height	Height Z-score Median (range)	Height cm Median (range)
Maternal (*n* = 45)	−0.9 (−2.8 to 0.8)	157.2 (154 to 168)
Paternal (*n* = 44)	−0.9 (−2.3 to 1.4)	169.9 (160 to 187)
Target height (*n* = 43)	−1 (−2 to 0.8)	170.4 (162 to 182.5)
Adult height (*n* = 23)	−0.9 (−2 to 0.8)	170.1 (162 to 182)

**Figure. 2:**
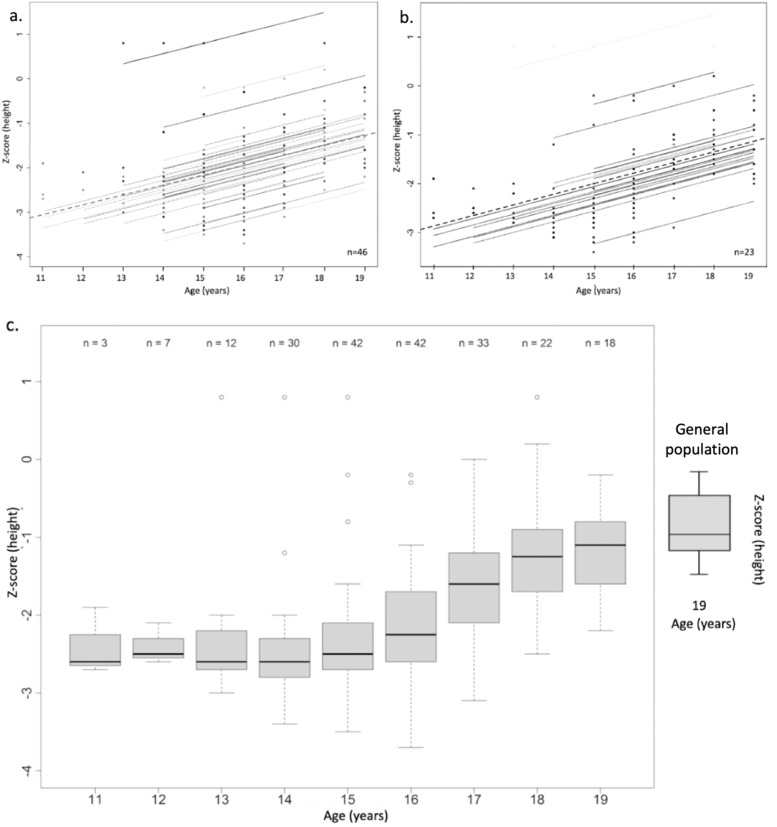
(a) Representation of height Z-score versus chronological age for all subjects. Each solid line represents the longitudinal growth trajectory of an individual patient (spaghetti plot). The dashed line represents the median Z-score at each age. Correlation coefficient of serial observations (95 % CI) = 0.74 (0.66; 0.80); *p* < 0.01. (b) Representation of height Z-score versus chronological age for the subgroup of subjects whose adult height is known. Each solid line represents an individual patient. The dashed line represents the median Z-score at each age. Correlation coefficient of serial observations (95 % CI) = 0.72 (0.61; 0.80); *p* < 0.01. (c) Box plot showing the evolution of height Z-score versus age, compared to the reference for Brazilian males at age 19 years. The leftmost box represents the reference value for the general population at adult height.

**Figure. 3:**
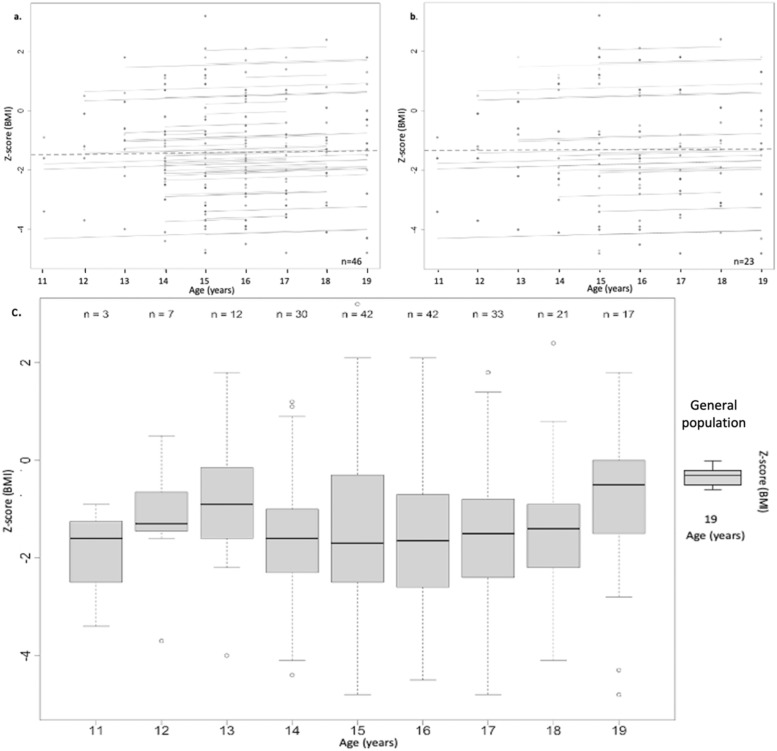
(a) Representation of BMI Z-score versus chronological age for all subjects. Each solid line represents the longitudinal trajectory of an individual patient (spaghetti plot). The dashed line represents the median Z-score at each age. Correlation coefficient of serial observations (95 % CI) = 0.11 (–0.05; 0.26); *p* = 0.18. (b) Representation of BMI Z-score versus chronological age for the subgroup of subjects whose adult height is known. Each solid line represents an individual patient. The dashed line represents the median Z-score at each age. Correlation coefficient of serial observations (95 % CI) = 0.08 (–0.11; 0.28); *p* = 0.40. (c) Box plot showing the evolution of BMI Z-score versus age, compared to the reference for Brazilian males at adult age. The leftmost box represents the reference value for the general population at adult BMI.

Follow-up until adult height was available for only 23 patients, as half of the cohort discontinued follow-up before reaching adulthood. Figures 2 and 3 illustrate the evolution and correlation coefficients of serial height and BMI observations, respectively.

In the first assessment of bone age, median chronological age was 14.9 (10.7 to 16.8), and median bone age delay was −2.5 SD (−5.6 to 0). In 82 % of cases, bone age was delayed. More than one evaluation of bone age was performed in 27 patients. In these cases, the last evaluation of 63 % of the patients still showed bone age delay. Median age was 17.4 (14.4 to 20), and median bone age delay was −2.2 (−2.3 to −0.6) SD.

**Figure. 4:**
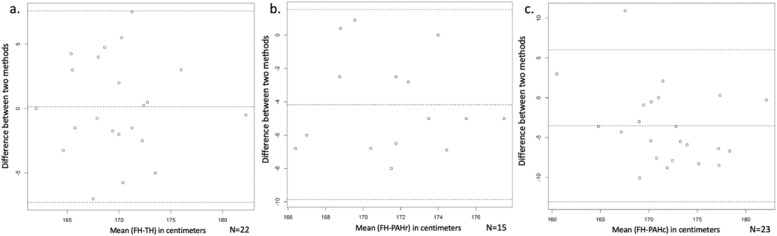
(a) Concordance between adult height (AH) and target height (TH). (b) Concordance between AH and predicted adult height (PAH) calculated using the Bayley-Pinneau table for delayed bone age. (c) Concordance between AH and PAH was calculated using the Bayley-Pinneau table for bone age within the expected range for puberty (concordant bone age). Each dot represents an individual patient. In all three panels, the central dashed horizontal line represents the median difference, and the outer dashed lines represent ±2 standard deviations from the median. The values on the vertical axis represent the difference in centimeters between the adult height and the predicted adult height according to each method.

The concordance between AH and PAH is shown in Figure 4. Considering bone age without delay, height was overestimated in 82 % of cases, with a median error of −4.3 (−10.1 to 10.9) centimeters. Considering the delayed bone age, height was overestimated in 80 % of cases, with a median error of −5 (−8 to 0.9) centimeters.

All patients experienced spontaneous puberty, with a median age of onset at 15 years (range: 14.5 to 16.7), and the median age at the end of puberty, available in 33 cases, was 17.2 years (range: 16 to 20). The median duration of puberty was 2.1 years (range: 1.2 to 4.8).

## Discussion

CDGP is the most common cause of DP in males, although it is a diagnosis of exclusion, necessitating vigilant monitoring during pediatric visits to discern and exclude pathological etiologies [4]. Once diagnosed, this condition is usually transient and has a benign evolution, with resolution of short stature, spontaneous pubertal development, and adaptation to the genetic height potential in most cases [2,3,5,6].

In this study, in >90 % of the patients, the complaint of delayed pubertal development was noticed by the family and confirmed by the physician. Vigilant anamnesis and physical examinations during pediatric consultations are imperative for timely detection. The favorable perinatal profiles of these patients, including term births and AGA, suggest a generally healthy variant of normal growth and development. Notably, congenital abnormalities such as bilateral cryptorchidism, micropenis, and hypospadias were absent, further supporting the benign nature of CDGP [5].

Family history of pubertal delay was present in 50 % of the cases – less than in studies with similar designs [5,6], but still relevant. It is important to note that, unfortunately, data on maternal menarche and paternal puberty were not found in most medical records. Although the cause of CDGP is unknown, it is known that there is a strong genetic influence, probably with an autosomal dominant pattern, with or without complete penetrance [5,10].

Delayed bone age, a hallmark of CDGP, was observed in the majority of cases, though its occurrence diminished with age progression.

Short stature resolved or became less important with age progression, and there was a good correlation between the increase in the height Z-score and time. The height of the patients whose AH is known is very close to the median height of the Brazilian man at age 19 years, according to Brazilian Institute of Geographics and Statistics (Instituto Brasileiro de Geografia e Estatística IBGE) data [21].

All patients whose AH was known reached their family channel, half of which was above and half below the target height. Approximately 82 % of the cases had a difference of up to five centimeters between the target height and the adult height. The prediction of AH using the Bayley-Pinneau method was less accurate and overestimated the adult height more frequently. Previous reviews comparing AH and PAH also found that PAH is overestimated by about 4 cm, very similar to this study. Another recent study that compared methods for adult height prediction suggests that the Roche-Wainer-Thissen (RWT) method may provide more accurate predictions in this population, particularly because it incorporates multiple variables beyond bone age alone, reducing the tendency to overestimate adult height in patients with significant bone age delay [6,22].

There was no correlation between the increase in the Z-score of BMI and the progression of chronological age in the population studied. In addition, classification of patients according to BMI was varied: most patients were eutrophic during the observations, but about one-third of them had a low BMI, without associated pathologies; few patients were overweight. These findings are compatible with the literature. Low weight and eutrophy predominate in cases of CDGP, although there is a portion of this population that may be overweight [8,9]. Interestingly, even as bone age delay and short stature tend to resolve over time, BMI remains relatively stable, with most patients maintaining a BMI within the eutrophic range throughout follow-up.

Puberty spontaneously started at around age 15 years – one year after the maximum limit adopted for the beginning of normal puberty, and ended at around age 17 years. Therefore, it had a duration close to puberty which develops in the usual period. This finding was expected and compatible with what has been found in previous studies [9]. Despite the benign and self-limited nature of CDGP, the psychological burden associated with delayed puberty, including low self-esteem and risk of bullying, is well recognized, particularly among boys. In such situations, short-term treatment with low doses of testosterone has traditionally been offered to accelerate the onset of pubertal changes and alleviate psychosocial distress. More recently, aromatase inhibitors, especially letrozole, have been investigated as alternative approaches, with the potential advantage of stimulating endogenous puberty while delaying epiphyseal maturation [12,13]. However, both therapeutic strategies should be reserved for carefully selected cases in which the psychosocial impact is significant.

The rate of abandonment of follow-up before the AH measurement was high: half of the cases. This observation may reinforce the hypothesis that CDGP is a variant of normality and has a good evolution, as described in other studies [6].

Limitations of the present study include its retrospective nature and reliance on incomplete medical records. The fact that CDGP is an extreme of normality and not a disease may have interfered with the low adherence to follow-up. Additionally, this sample represents a population referred to a tertiary center, which may differ from the general population of individuals with CDGP, as many of them do not require specialized care. Nevertheless, a key strength of this study is its real-world setting, reflecting a historical cohort of patients who were managed without testosterone or somatropin treatment. Furthermore, all patients were followed in the same center and evaluated by the same group of physicians, ensuring consistency in clinical assessment.

In conclusion, the evolution of height and pubertal development in patients with CDPG is good. Their height development occurs within their genetic potential, despite the fact that puberty onset and attainment of AH occur later in comparison to the population average. AH prediction according to the Bayley-Pinneau method overestimated AH, while their AH was closely related to TH.

The present findings highlight the benign course of CDGP, characterized by spontaneous pubertal progression and attainment of adult height generally consistent with genetic potential, without the need for growth-promoting therapies. This reinforces that CDGP is a normal variant of pubertal timing rather than a pathological condition. Continued physician awareness is essential to differentiate CDGP from pathological causes of pubertal delay and to avoid unnecessary treatments. Further research is needed to improve the understanding and management of CDGP and other growth variations.

## Conflicts of interest

The authors declare no conflicts of interest.
